# Long noncoding RNA *GAS5* attenuates cigarettesmoke-induced airway remodeling by regulatingmiR-217-5p/PTEN axis: *GAS5* attenuates airway remodeling by miR-217-5p/PTEN axis

**DOI:** 10.3724/abbs.2022074

**Published:** 2022-06-17

**Authors:** Yong Du, Yi Ding, Tianyun Shi, Wei He, Jingjing Feng, Zhoufang Mei, Xuru Chen, Xintong Feng, Xiaohua Zhang, Zhijun Jie

**Affiliations:** Department of Pulmonary and Critical Care Medicine Shanghai Fifth People’s Hospital Fudan University Shanghai 200240 China

**Keywords:** airway remodeling, COPD, GAS5, miR-217-5p, PTEN

## Abstract

Airway remodeling is a remarkable pathological characteristic of chronic obstructive pulmonary disease (COPD), and long noncoding RNAs have been demonstrated to participate in COPD development and pathogenesis. Here, we investigate the role of long noncoding RNA *GAS5* in cigarette smoke (CS)-induced airway remodeling. *GAS5* expression is significantly lower in lung tissues of CS-exposed mice than in tissues of control mice without exposure to CS. Forced *GAS5* overexpression suppresses CS-induced airway inflammation and remodeling. *GAS5* overexpression also inhibits CS extract-induced inflammatory-cytokine expression and fibroblast activation *in vitro*. Regarding the mechanism, *GAS5* acts as a sponge of miR-217-5p, thereby increasing PTEN expression. MiR-217-5p overexpression and PTEN knockdown separately reverse the inhibitory effects of *GAS5* overexpression on the inflammatory-cytokine expression and fibroblast activation. Collectively, these results suggest that *GAS5* can suppress airway inflammation and fibroblast activation by regulating miR-217-5p/PTEN axis, which may help develop novel therapeutic strategies against COPD.

## Introduction

Chronic obstructive pulmonary disease (COPD) is a widespread, preventable, and curable disease characterized by multiple respiratory signs and gradual airflow obstruction due to airway or alveolar impairments typically induced by destructive inhaled gases and noxious inflammatory particles [1]. COPD doubles the risk of cardiovascular disease and is the fourth most common cause of mortality globally [2]. Throughout COPD progression, tobacco smoking is the most important risk factor of this disease and contributes to structural remodeling of the airways [3]. Airway remodeling as a result of exposure to cigarette smoke (CS) leads to small-airway contraction and has been linked to the differentiation of bronchial fibroblasts into myofibroblasts, including massive deposition of the extracellular matrix [4]. Nevertheless, the specific molecular mechanisms underlying significant airway remodeling reactions to CS are not fully understood.

Long noncoding RNAs (lncRNAs) are non-protein-coding RNAs with length over 200 nucleotides [5]. LncRNAs have been found in multiple theoretical models as controllers of gene expression regulators; consequently, they may play crucial roles in many biological processes and in disease management [6]. Aberrant expression levels of lncRNAs have been implicated in the pathogenesis and development of COPD [7]. For example, lncRNA *MIR155HG* expression levels is increased in the lung parenchyma of smokers with COPD and in cigarette smoke extract (CSE)-treated human pulmonary microvascular endothelial cells in a dose- and time-dependent manner [8]. The levels of lncRNA *TUG1* are significantly higher in COPD patients and CSE-treated BEAS-2B cells than in controls. LncRNA *TUG1* depletion improves cell proliferation but inhibits apoptosis and inflammatory reactions of CSE-treated BEAS-2B cells [9]. Shen *et al*. [10] reported that *SNHG5* overexpression attenuates the effects of CSE on cellular proliferation, inflammatory processes, and apoptosis; moreover, CSE can decrease lncRNA *SNHG5* expression in 16HBE cells. Another lncRNA, growth arrest-specific 5 ( *GAS5*), may affect various respiratory diseases, including lung cancer, asthma, and fibrosis [ 10– 12] ; however, there is no study on its mechanisms of action and involvement in CS-caused airway remodeling.

In the present study, we hypothesized that *GAS5* may attenuate CS-induced airway remodeling by repressing airway inflammation and lung fibroblast activation. Accordingly, we assessed the expression of *GAS5* in CS-exposed mice and investigated the mechanisms of *GAS5* action in airway remodeling in cultured cells and in laboratory mice.

## Materials and Methods

### Lentivirus production

*GAS5*-overexpressing lentivirus and control lentivirus (empty vector) were designed and assembled by GenePharma (Shanghai, China). MiR-217-5p mimic (5′-UACUGCAUCAGGAACUGACUGGA-3′) and its negative control (NC, 5′-UUCUCCGAACGUGUCACGUTT-3′) mimic were purchased from RiboBio (Guangzhou, China). To knock down PTEN, a specific small interfering RNA (siRNA) targeting *PTEN* mRNA (siPTEN, 5′-AGGTGAAGATATATTCCTCCAA-3′) and scramble siRNA (control, 5′-UUCUCCGAACGUGUCACGU-3′) were designed and synthesized.

### CS exposure

All animal experiment procedures were approved by the Animal Research Committee at Shanghai Fifth People’s Hospital. C57BL/6 male mice (20–25 g; SLAC Laboratory, Shanghai, China) were maintained at a 12/12 h light/dark cycle in an animal facility at 21–24°C. Water and conventional mouse feed were available to the animals *ad libitum*. The mice were randomly assigned into experimental and control groups and allowed to acclimate substantially for 1 week before the experiments. The whole body of each mouse in the experimental group was exposed to CS, as outlined previously [13]. Briefly, the mice were exposed four times per day to nicotine-containing smoke from five tobacco plants, for 30 min each time. The mice were exposed to CS 5 days a week for 24 weeks. The control mice were not exposed to CS in the presence of room air only. Subsequent pathophysiological analyses were completed 24 weeks after the CS exposure.

### Animal experiments

Mice were subdivided into four groups, i.e., standard control group, CS+empty vector control group, CS group, and CS+ *GAS5* overexpression group ( *n*=5 per group). Mice in the *GAS5* overexpression group and empty-vector control group were injected with the corresponding lentivirus through the tail vein (2×10 ^7^ TU in 50 μL) once every 2 weeks starting from week 2 post CS exposure. The CS exposure was performed as described above. The mice were euthanized and analyzed the day after the latest CS exposure together with the corresponding control mice without exposure to CS.

### Histological staining

After excision, the lung parenchyma of mice was fixed immediately in 10% formalin and then sliced into paraffin-embedded sections. The tissue sections were processed for subsequent hematoxylin and eosin (H&E) staining, Masson staining, and α-SMA immunohistochemical analysis as previously reported [11].

### Collection of bronchoalveolar lavage fluid (BALF)

The lung parenchyma was perfused with phosphate-buffered saline (PBS) after both the thorax and trachea were exposed. PBS (500 μL) was injected into and collected from the trachea twice, and this procedure was performed three times on all mice. The resultant liquid was centrifuged at 1500 *g* for 10 min. Cytospin slides were subject to Wright-Giemsa staining [14]. In a blinded procedure, all cells and neutrophils in the BALF were counted under a microscope.

### RNA isolation and quantitative reverse-transcription PCR

Total mRNA was isolated from tissues and cells using the Trizol reagent (Invitrogen, Carlsbad, USA). Complementary DNA was prepared using the Prime Script 1 ^st^ Strand cDNA Synthesis kit (TaKaRa, Dalian, China). Expression levels of *GAS5*, *IL6*, *TNFA*, and *TGFB* were determined by qPCR using the SYBR Green PCR Master Mix (Applied Biosystems, Stockholm, Sweden) with the following cycling conditions: 95°C for 30 s, followed by 40 cycles of 95°C for 10 s, and 60°C for 30 s. The relative expression of each transcript was calculated using the 2 ^–ΔΔCT^ method, and the mRNA level of each gene was normalized to that of *GAPDH*. Each sample was assayed in triplicate. The PCR primers for *GAS5* were 5′-AGCTGGAAGTTGAAATGG-3′ (forward) and 5′-CAAGCCGACTCTCCATAC-3′ (reverse). The primers for *IL6* were 5′-CCAGAAACCGCTATGAAGTTCC-3′ (forward) and 5′-GTTGGGAGTGGTATCCTCTGTGA-3′ (reverse). The primers for *TNFA* were 5′-GCAACTGCTGCACGAAATC-3′ (forward) and 5′-CTGCTTGTCCTCTGCCCAC-3′ (reverse). The primers for *TGFB* were 5′-CCGCAACAACGCCATCTATG-3′ (forward) and 5′- CCCGAATGTCTGACGTATTGAAG-3′ (reverse). The primers for *GAPDH* were 5′-GGTCTCCTCTGACTTCAACA-3′ (forward) and 5′- GTGAGGGTCTCTCTCTTCCT-3′ (reverse).

### Western blot analysis

Cells were lyzed in RIPA lysis buffer containing a protease inhibitor cocktail. Lysate total protein was quantified using a BCA Protein Assay kit (Thermo Scientific, Rockford, USA). Proteins were separated by electrophoresis in an 8%–10% SDS-polyacrylamide gel and then transferred onto a polyvinylidene fluoride membrane. Nonspecific binding sites on the membranes were blocked with a solution of powdered milk (5%) at room temperature for 30 min, and then the membranes were incubated with primary antibodies against α-SMA (Proteintech, Wuhan, China), tubulin (Proteintech), collagen I (Proteintech), and PTEN (Cell Signaling Technology, Danvers, USA) at 4°C overnight. After 30 min of incubation with a corresponding horseradish peroxidase-conjugated secondary antibody at room temperature, protein bands on each membrane were identified using a chemiluminescent response in the presence of a horseradish peroxidase substrate (Beyotime, Nantong, China) and quantified using ImageJ software. Tubulin was used as the loading control.

### Enzyme-linked immunosorbent assay (ELISA)

Lungs were homogenized and sonicated in PBS containing protease inhibitors. Homogenates were centrifuged at 900 *g* for 15 min. The levels of mouse IL6, TNF-α and TGF-β1 in lung tissue homogenates and BALF were quantified using ELISA kits obtained from Cloud-Clone Corporation (Wuhan, China), respectively, according to the manufacturer’s instructions.

### Preparation of cigarette smoke extracts

The smoke from 10 3R4F reference cigarettes was bubbled through 30 mL of a medium to prepare the CSE. The resulting solution was filter-sterilized and designated as 100% CSE. The CSE suspension was diluted with the culture medium to a final concentration of 2.5% and kept at –80°C until use.

### Cell culture and treatments

Murine alveolar epithelial cell line MLE-12 was purchased from the American Type Culture Collection (ATCC; Manassas, USA). MLE-12 cells were cultured in the RPMI 1640 medium supplemented with 10% of fetal bovine serum (FBS) in a humidified atmosphere at 37°C with 5% CO _2_. Mouse primary lung fibroblasts were isolated as described previously [15]. Mouse lung tissues containing small airways was minced into 1–2 mm pieces, digested with 0.28 U/mL liberase blendzyme 3 (Sigma, St Louis, USA) and 60 U/mL DNase I (Sigma) for 45 min at 37°C in medium, passed through a 70-μm filter, centrifuged at 540 *g* at 4°C, and plated in tissue culture flasks in Dulbecco’s modified Eagle’s medium supplemented with 15% FBS. Cells were passaged when subconfluent after harvest with trypsin-EDTA. Cells were used for experiments at passages 3 and 4. The cells were exposed to 2.5% CSE for 24 h after reaching 70%–80% confluence.

### Vector construction and luciferase reporter assay

Plasmids were constructed from fragments of the *PTEN* 3′untranslated region (3′UTR) and of the *GAS5* gene including either a wild-type (wt) or mutant (mut) binding site for miR-217-5p. The DNA fragments were inserted into the pmirGLO vector. The miR-217-5p mimic, its NC, and luciferase reporter plasmids were cotransfected into MLE-12 cells. The plasmids with mut binding sites served as the control. A Dual-Glo Luciferase Assay System (Promega, Madison, USA) was employed to determine the ratio of firefly luciferase–generated luminescence to *Renilla* luciferase-generated luminescence, *i.e.*, relative luciferase activity.

### Statistical analysis

GraphPad Prism Software 5 was utilized for statistical analysis. Data are presented as the mean±SD from at least three independent experiments. Student’s *t* test or analysis of variance was performed to determine statistical significance, and *P*<0.05 was deemed statistically significant.

## Results

### *GAS5* attenuates airway remodeling in CS-exposed mice

**Figure 1:**
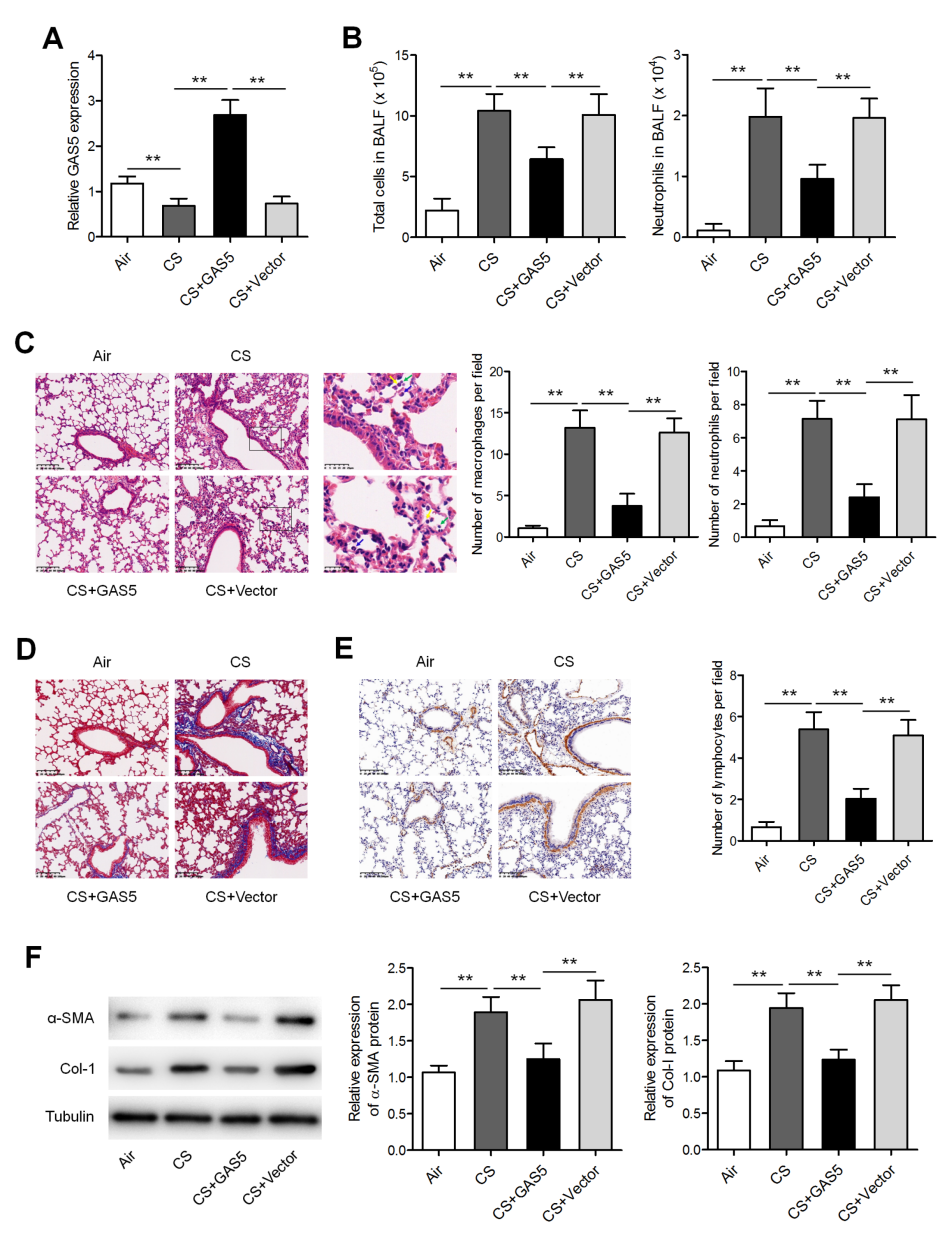
Figure 1 ***GAS5*** **attenuated CS-induced airway remodeling** (A) The expression of GAS5 was measured by qRT-PCR in the lungs of mice with or without exposure to CS and injected with either a GAS5-overexpressing or an empty (control) lentiviral vector. Vector: empty vector. (B) The numbers of total cells and neutrophils in the BALF from the mice. (C) Histological analysis of the lung slices was performed by H&E staining. Average numbers of inflammation cells per field of view were also shown. Scale bar: 100 μm. (D) Morphology of the mouse lung slices was evaluated by Masson staining. Scale bar: 100 μm. (E) Lung slices were assessed by α-SMA staining. Scale bar: 100 μm. (F) Western blot analysis of the protein levels of α-SMA and collagen I (Col-I) in lungs and BALF, respectively. ** P<0.01.

**Figure 2:**
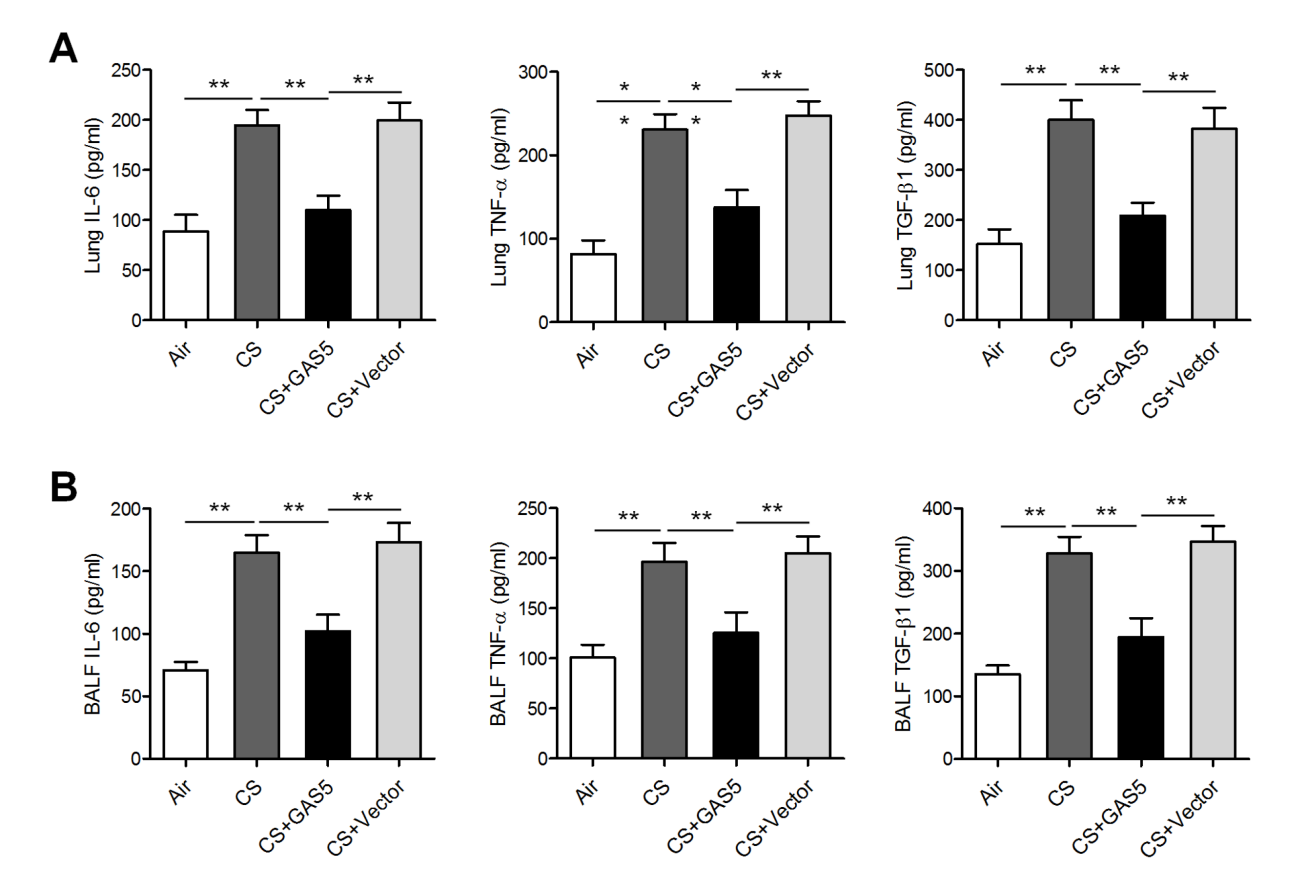
Figure 2 ***GAS5*** **attenuated CS-induced airway inflammatory processes** (A) Enzyme-linked immunosorbent assays of IL-6, TNF-α, and TGF-β1 in the lung homogenate of mice with or without exposure to CS and injected with either the GAS5-overexpressing or empty (control) lentiviral vector. Vector: empty vector. (B) Enzyme-linked immunosorbent assays of IL-6, TNF-α, and TGF-β1 in BALF. * P<0.05, ** P<0.01.

To ascertain whether *GAS5* is associated with the pathogenesis of airway remodeling, the expression of *GAS5* was measured in a mouse model of CS exposure. qRT-PCR results revealed that *GAS5* expression was significantly lower in the lung parenchyma of CS-exposed mice compared to that in control mice without exposure to CS; Figure 1A). The lentivirus overexpressing *GAS5* was injected through the tail concurrently with the CS exposure. *GAS5* was found to be upregulated in the lungs of the mice that received the *GAS5-*overexpressing lentivirus, confirming the successful increase in *GAS5* levels ( Figure 1A). The numbers of all cells and of neutrophils were found to be elevated in the BALF from CS-exposed mice ( Figure 1B). In the CS-exposed mice with forced *GAS5* overexpression, H&E-staining of lung sections showed a reduction in peribronchial infiltrates of inflammatory cells ( Figure 1C). As evidenced by immunohistochemical analysis, parameters of airway remodeling, including smooth muscle mass and peribronchial collagen deposition, were significantly decreased in the CS-exposed mice with *GAS5* overexpression compared to CS-exposed mice without this overexpression ( Figure 1D,E). Similarly, western blot analysis revealed that the *GAS5* overexpression reduced the expressions of both α-SMA and collagen I in CS-exposed mice ( Figure 1F). Moreover, the expressions of proinflammatory factors involved in COPD pathogenesis, such as IL6, TNF-α, and TGF-β1, were found to be significantly inhibited in both the lung homogenates and BALF from CS-exposed mice after *GAS5* overexpression ( Figure 2).

### *GAS5* weakens CSE-induced inflammatory-cytokine expression and fibroblast activation

**Figure 3:**
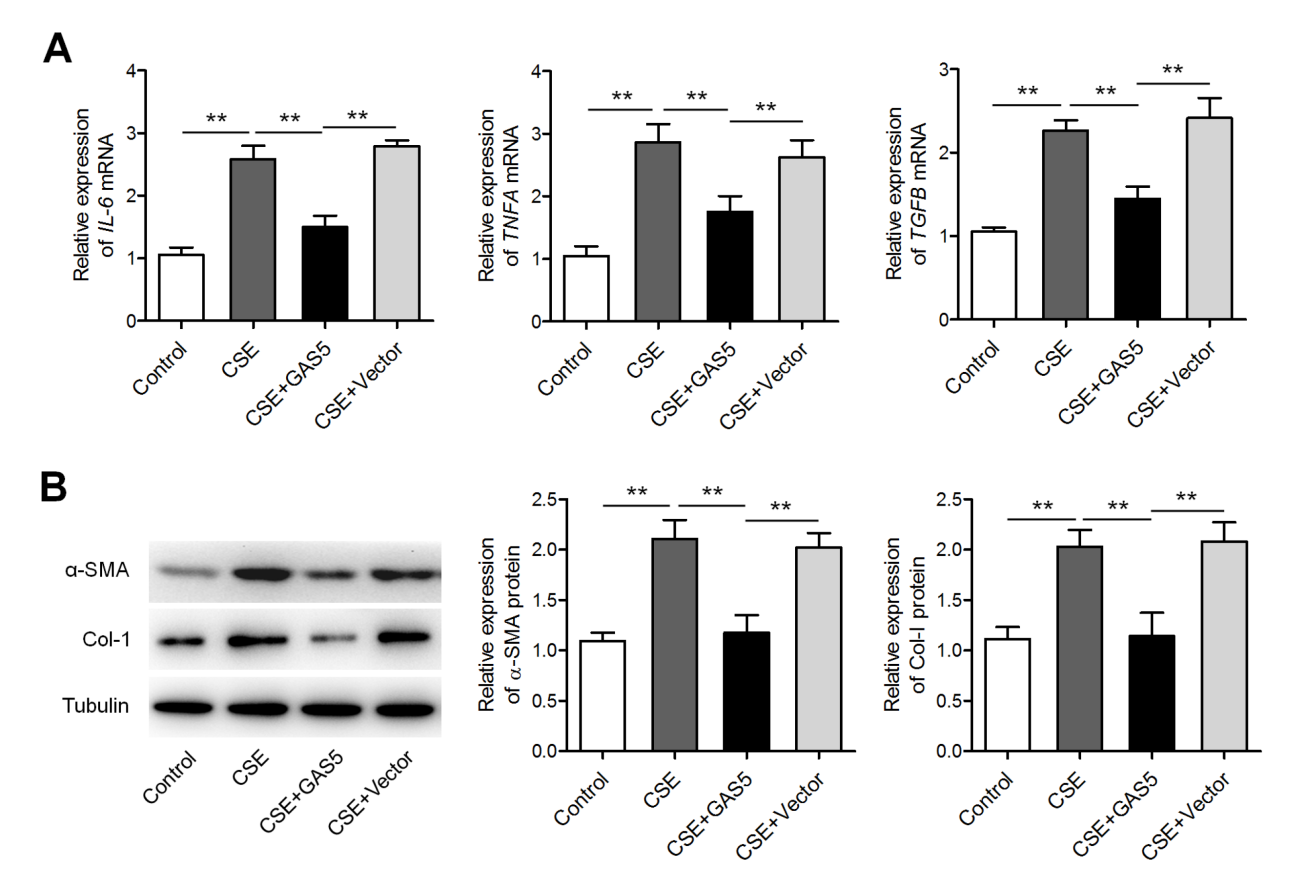
Figure 3 ***GAS5*** **attenuated CSE-induced inflammatory-cytokine expression and fibroblast activation** (A) qRT-PCR analysis of the expressions of IL6, TNFA, and TGFB mRNAs in 2.5% CSE-treated MLE-12 cells transfected with either the GAS5-overexpressing or empty (control) lentiviral vector. Vector: empty vector. (B) Western blot analysis of the protein levels of α-SMA and collagen I in murine lung fibroblasts treated with 2.5% CSE after transfection with either the GAS5-overexpressing or control lentiviral vector. ** P<0.01.

Next, murine alveolar epithelial cells (MLE-12 cell line) were infected with the lentivirus carrying *GAS5*. As shown in Figure 3A, 2.5% CSE treatment promoted the expressions of *IL6*, *TNFA*, and *TGFB* mRNAs, whereas *GAS5* overexpression attenuated these effects. Furthermore, given that fibroblasts constitute a significant source of airway fibrosis and extracellular-matrix protein deposition, thereby leading to peripheral airway contraction in COPD [16], we tested whether *GAS5* performs important function in parenchymal fibroblasts under these circumstances. In murine fibroblasts, overexpression of *GAS5* significantly weakened the CSE-induced upregulation of α-SMA and collagen I ( Figure 3B).

### *GAS5* acts as a sponge of miR-217-5p and thereby increases PTEN expression

**Figure 4:**
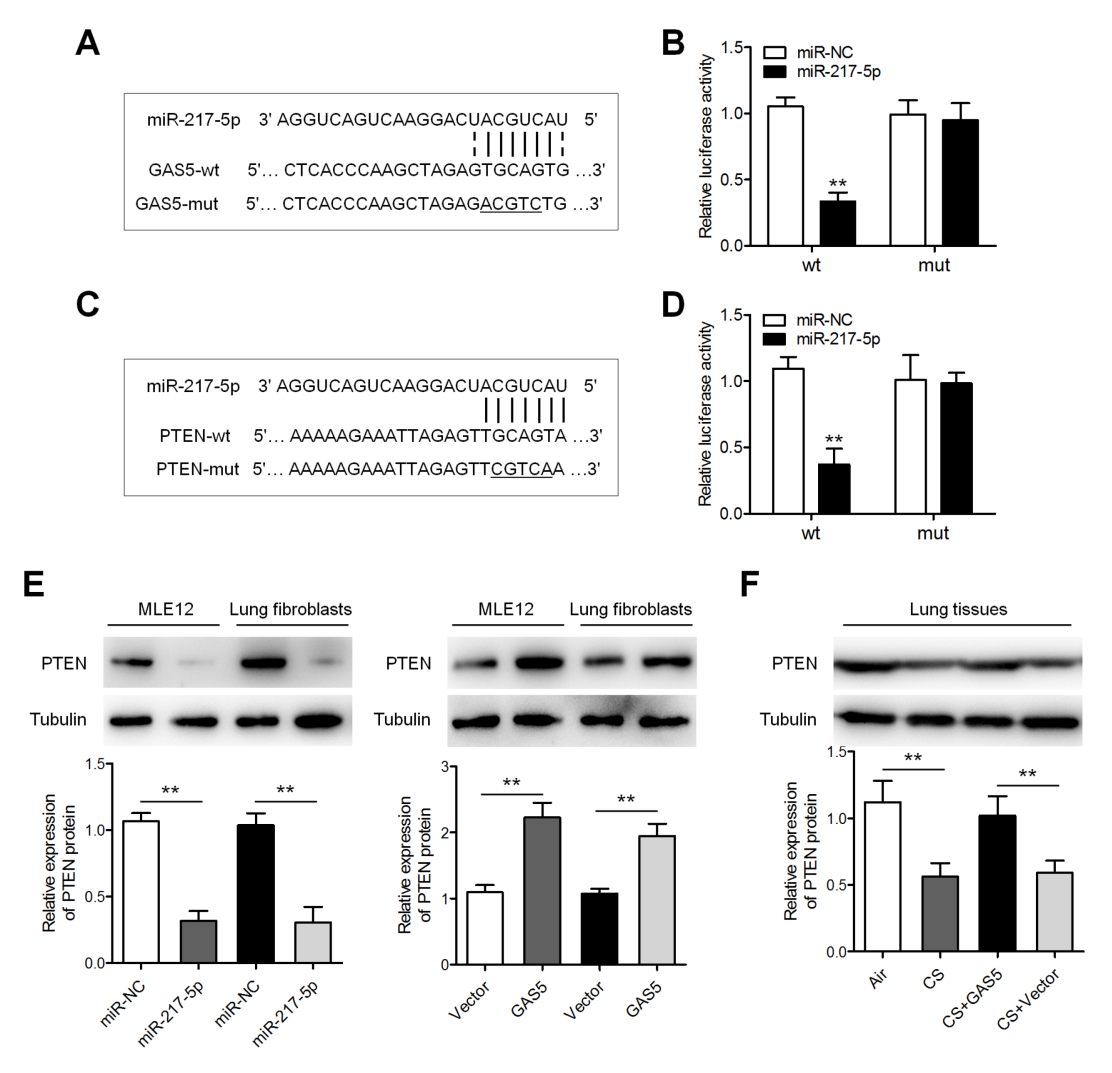
Figure 4 ***GAS5*** **increased PTEN expression by sponging miR-217-5p** (A) The predicted miR-217-5p-binding sequence in GAS5. (B) Estimated luciferase activity. (C) The predicted miR-217-5p–binding sequence in the PTEN 3′UTR. (D) Reassessment of the relative luciferase activity. (E) Expression of PTEN protein detected by western blot analysis in MLE-12 cells and murine lung fibroblasts after transfection with either the GAS5-overexpressing lentivirus or the miR-217-5p mimic. Vector: empty vector. (F) Western blot analysis of PTEN expression in the lungs of the mice with or without exposure to CS and injected with either the GAS5-overexpressing or control lentiviral vector. ** P<0.01.

To illustrate the molecular mechanisms by which *GAS5* exerts its effect on COPD, we screened potential targets of *GAS5* and found that miR-217-5p has a binding site in *GAS5* ( Figure 4A). To verify whether miR-217-5p is the primary target of *GAS5*, reporter plasmids bearing a *GAS5* gene fragment with either a wt or mut binding site for miR-217-5p were constructed, which were named GAS5-wt and GAS5-mut, respectively. Cotransfection of the miR-217-5p mimic and GAS5-wt plasmid significantly reduced luciferase activity, whereas cotransfection of the miR-217-5p mimic and GAS5-mut did not alter luciferase activity ( Figure 4B). *PTEN*, which can ameliorate lung fibrosis and inflammation, has been reported to be a primary target gene of miR-217-5p [ 17– 19] . We then conducted a luciferase assay which confirmed that the miR-217-5p mimic represses the luciferase activity of the *PTEN* 3′UTR-wt plasmid but not the luciferase activity of the *PTEN* 3′UTR-mut plasmid ( Figure 4C,D). In MLE-12 cells and murine lung fibroblasts, western blot analysis indicated that the miR-217-5p mimic significantly inhibited the expression of PTEN, whereas *GAS5* enhanced PTEN expression ( Figure 4E,F). Moreover, *GAS5* caused a notable increase in PTEN expression in the CS-exposed mice.

### *GAS5* exerts its effects by increasing miR-217-5p/PTEN axis output

**Figure 5:**
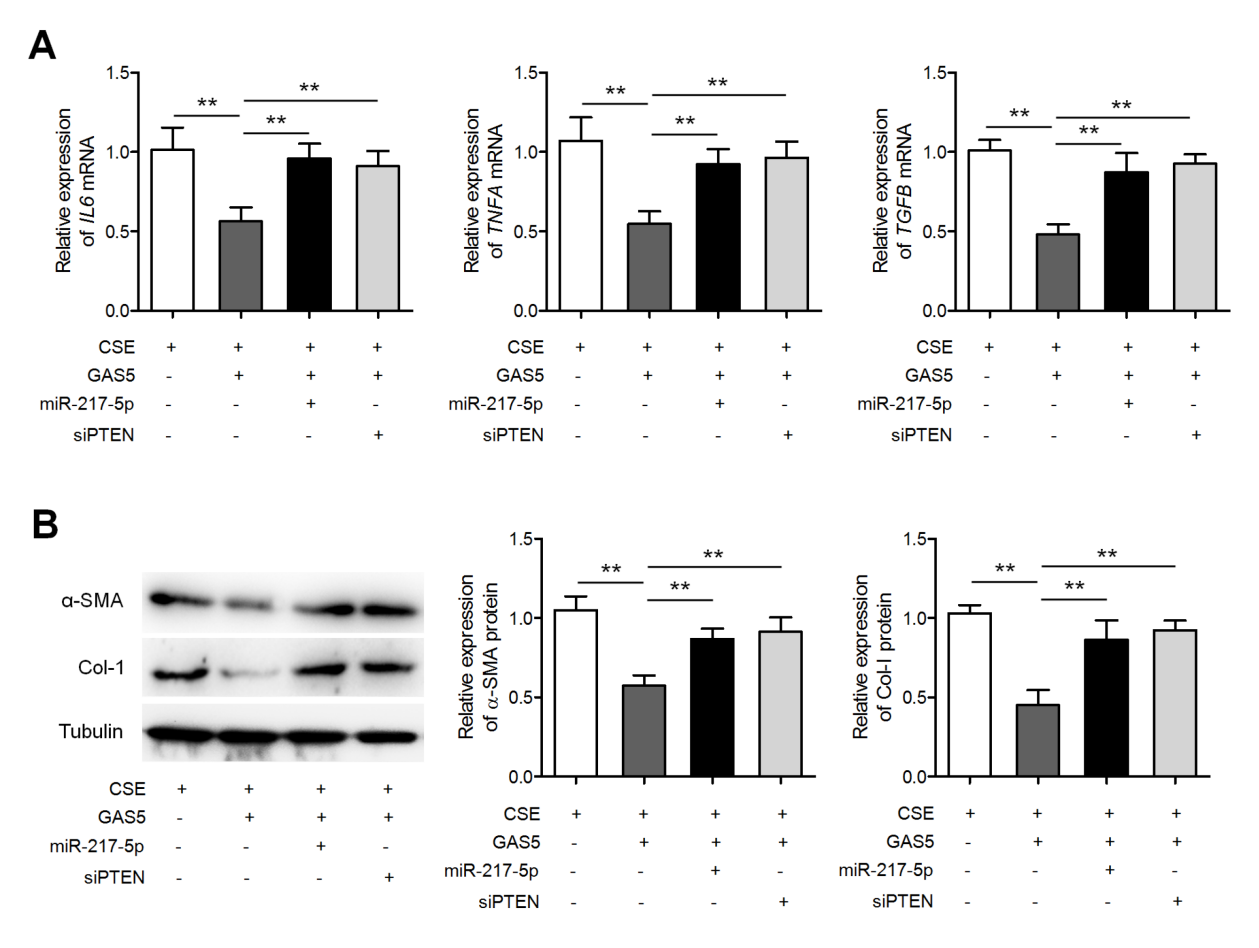
Figure 5 **MiR-217-5p overexpression and PTEN knockdown separately reversed the** ***GAS5***-driven inhibition of airway inflammation and fibrosis (A) GAS5-overexpressing MLE-12 cells were transfected with either the miR-217-5p mimic or PTEN siRNA (siPTEN) followed by treatment with 2.5% CSE. The expressions of IL6, TNFA, and TGFB mRNAs were measured by qRT-PCR. (B) GAS5-overexpressing fibroblasts were transfected with either the PTEN siRNA or miR-217-5p mimic, followed by CSE treatment. Both α-SMA and collagen I were quantified by western blot analysis. ** P<0.01.

**Figure 6:**
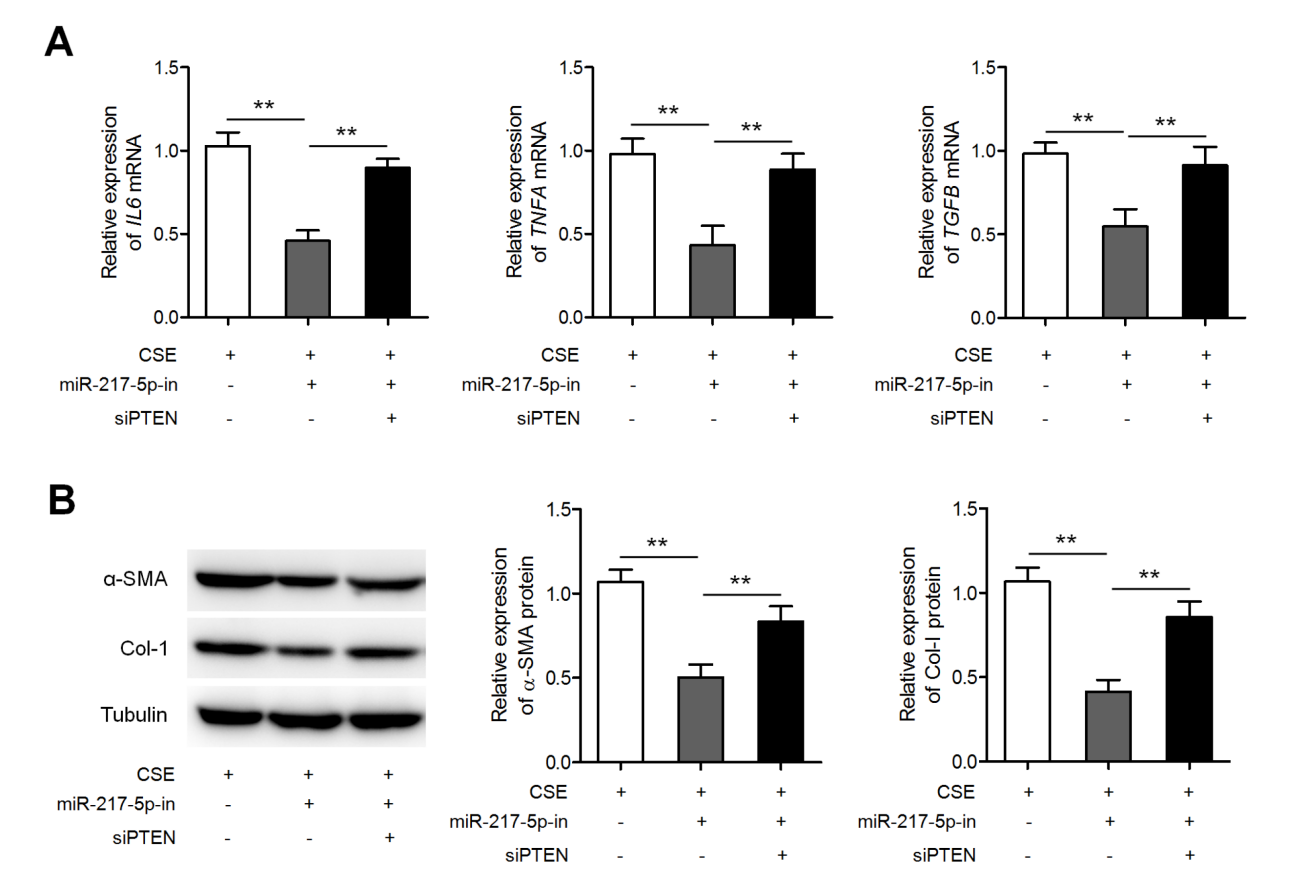
Figure 6 MiR-217-5p knockdown suppressed CSE-induced inflammation and fibroblast activation (A) MLE-12 cells were transfected with either the miR-217-5p inhibitor or siPTEN, followed by treatment with 2.5% CSE. The expressions of IL6, TNFA, and TGFB mRNAs were measured by qRT-PCR. (B) Western blot analysis of α-SMA and collagen I expressions in these cells. ** P<0.01.

Finally, we investigated whether miR-217-5p and PTEN mediate the beneficial inhibitory effects of *GAS5* on CSE-induced inflammatory-cytokine expression and fibroblast activation. Figure 5A shows that miR-217-5p overexpression and PTEN knockdown separately upregulated *IL6*, *TNFA*, and *TGFB* expressions in *GAS5*-overexpressing MLE-12 cells during CSE treatment. Meanwhile, miR-217-5p overexpression and PTEN knockdown separately enhanced the expressions of α-SMA and collagen I in *GAS5*-overexpressing fibroblasts during CSE treatment ( Figure 5B). Furthermore, miR-217-5p inhibitor suppressed CSE-induced upregulation of *IL6*, *TNFA*, and *TGFB* mRNAs ( Figure 6A), as well as α-SMA and collagen I proteins ( Figure 6B), and these effects could be reversed by siPTEN. These results suggest that *GAS5* decreases inflammatory cell responses and fibroblast activation by increasing the output of the miR-217-5p–PTEN pathway.

## Discussion

Airway remodeling in COPD is a complex pathophysiological process that involves inflammation, fibrosis, peribronchial collagen deposition, oxidative stress and other pathological changes, which eventually leads to irreversible and progressive airflow limitation [20]. CS exposure-induced airway remodeling leads to the narrowing of the small airways, where bronchial epithelium and fibroblasts are involved. Bronchial epithelial cells, a first-line contact with harmful substances, are a source of various cytokines, chemokines, and factors that modulate mesenchymal fibroblasts and smooth muscle cells and recruit immune cells in response to CS. In the present study, we investigated the function of lncRNA *GAS5* in CS-induced airway remodeling. We found that *GAS5* expression was significantly lower in the lung tissues of CS-exposed mice compared with that in the control mice without exposure to CS. Overexpression of *GAS5* inhibited airway remodeling in CS-exposed mice and similarly weakened CSE-induced expressions of inflammatory cytokines and fibroblast activation *in vitro*, suggesting a protective effect against inflammation and fibrosis in COPD. These data are consistent with the findings of Shan *et al*. [21] who, by using only *in vitro* assays, showed that *GAS5* weakens inflammatory effects and apoptosis of alveolar epithelial cells by targeting the miR-429/DUSP1 axis.

LncRNAs can influence mRNA translation by acting as miRNA sponges, thereby modulating various biological functions. Growing evidence has revealed that lncRNAs and miRNAs perform crucial functions in the pathogenesis and progression of COPD [21]. By serving as a miR-132 sponge and by enhancing the expression of its target ( *PTEN* mRNA), overexpressed lncRNA *SNHG5* in 16HBE cells attenuates the effects of CSE on cell proliferation, apoptosis, and inflammatory responses [22]. By targeting miR-218, lncRNA *MEG3* reverses CSE-repressed cell proliferation, CSE-induced apoptosis and inflammatory processes [23]. By regulating the miR-128-5p/BRD4 axis, lncRNA *MIR155HG* can induce apoptosis and inflammatory responses of human pulmonary microvascular endothelial cells in smoke-related COPD [8]. LncRNA *GAS5* can also serve as a molecular sponge of miRNAs in many diseases. For example, *GAS5* silencing protects H9c2 cardiomyocytes against hypoxia-induced damage by sponging miR-142-5p and thereby functionally liberating *TP53INP1* mRNA transcripts [24]. By operating as a competing endogenous RNA of miR-96-5p, *GAS5* intensifies renal tubular epithelial fibrosis [25]. By means of the miR-455-5p/SOCS3 pathway, *GAS5* boosts M1 macrophage polarization in adolescent pneumonia [26]. In the present study, bioinformatics software was employed to identify miR-217-5p as a new target of *GAS5*, and this finding was then confirmed by luciferase reporter assays.

MiR-217-5p is encoded by human chromosome 2 and mouse chromosome 11, and is conserved among humans, mice, and rats [19]. Recently, numerous studies have confirmed that miR-217 exerts diverse actions in physiological and pathological processes. For example, aging-associated miR-217 exacerbates atherosclerosis and promotes cardiovascular dysfunction, thereby highlighting a therapeutic potential of miR-217 inhibitors in aging-related cardiovascular diseases [27]. MiR-217 is known to be upregulated in murine lungs during sepsis, and miR-217 inhibition protects against lung injury and improves the survival rate of septic mice [28]. Consistent with these findings, our study indicates that miR-217-5p may enhance inflammation and fibrosis; that is, miR-217-5p overexpression reverses the inhibitory effects of *GAS5* on CSE-induced inflammatory-cytokine expression and fibroblast activation. It is worth noting that by downregulating PTEN, miR-217 partakes in cardiac fibrosis and cardiac hypertrophy processes [19]. Here, by luciferase reporter assays, we demonstrated that miR-217-5p mimic inhibited the luciferase activity of the *PTEN* 3′UTR-wt plasmid, but not that of the *PTEN* 3′UTR-mut plasmid. In addition, the miR-217-5p mimic significantly reduced PTEN expression in both MLE-12 cells and murine lung fibroblasts. These findings suggest that miR-217-5p can directly target the 3′UTR of *PTEN* mRNA and suppress PTEN expression.

PTEN is a well-characterized negative regulator of PI3K which transforms phosphatidylinositol-3,4,5-phosphate (PIP3) to phosphatidylinositol-4,5-phosphate (PIP2), thus promoting the inactivation of phosphoinositide-dependent kinase 1 (PDK1) and of its target AKT. Initially, *PTEN* was identified as a tumor suppressor gene that encodes a phosphatase implicated in the inactivation of maturation and differentiation [ 17– 19] . PTEN has been shown to be often mutated or even absent in the epithelia of patients with lung cancer and in smokers [ 17– 19] . In COPD patients, PTEN protein level was found to be diminished by oxidative stress, resulting in persistent activation of both the PI3K-AKT pathway and a proinflammatory mediator release. Hence, improving the function of anti-inflammatory PTEN may be a promising therapeutic strategy against COPD development [18]. Here, we found that *GAS5* enhances PTEN expression in MLE-12 cells and murine lung fibroblasts. Accordingly, *GAS5* caused a notable increase in PTEN expression in the lungs of CS-exposed mice. Furthermore, miR-217-5p overexpression and PTEN knockdown separately reverse *GAS5*-driven inhibition of airway inflammation and fibrosis, suggesting that *GAS5* attenuates inflammation and fibrosis by upregulating the miR-217-5p/PTEN axis output.

In conclusion, our results indicate that *GAS5* may suppress CS-induced airway remodeling by increasing the output of the miR-217-5p/PTEN pathway. This study may help develop novel therapeutic strategies against COPD progression.
